# From TVs to tablets: the relation between device-specific screen time and health-related behaviors and characteristics

**DOI:** 10.1186/s12889-020-09410-0

**Published:** 2020-08-26

**Authors:** Maricarmen Vizcaino, Matthew Buman, Tyler DesRoches, Christopher Wharton

**Affiliations:** 1 Radical Simplicity Lab, College of Health Solutions, Arizona State University, 550 N 3rd Street, Phoenix, AZ 85004 USA; 2 College of Health Solutions, Arizona State University, Tempe, USA; 3 School of Sustainability/ School of Historical, Philosophical and Religious Studies, Arizona State University, Tempe, USA

**Keywords:** Screen-time, Television, Diet, Dietary choices, Dietary habits, Health, Stress

## Abstract

**Background:**

The purpose of this study was to examine whether extended use of a variety of screen-based devices, in addition to television, was associated with poor dietary habits and other health-related characteristics and behaviors among US adults. The recent phenomenon of binge-watching was also explored.

**Methods:**

A survey to assess screen time across multiple devices, dietary habits, sleep duration and quality, perceived stress, self-rated health, physical activity, and body mass index, was administered to a sample of US adults using the Qualtrics platform and distributed via Amazon Mechanical Turk (MTurk). Participants were adults 18 years of age and older, English speakers, current US residents, and owners of a television and at least one other device with a screen. Three different screen time categories (heavy, moderate, and light) were created for total screen time, and separately for screen time by type of screen, based on distribution tertiles. Kruskal-Wallis tests were conducted to examine differences in dietary habits and health-related characteristics between screen time categories.

**Results:**

Aggregate screen time across all devices totaled 17.5 h per day for heavy users. Heavy users reported the least healthful dietary patterns and the poorest health-related characteristics – including self-rated health – compared to moderate and light users. Moreover, unique dietary habits emerged when examining dietary patterns by type of screen separately, such that heavy users of TV and smartphone displayed the least healthful dietary patterns compared to heavy users of TV-connected devices, laptop, and tablet. Binge-watching was also significantly associated with less healthy dietary patterns, including frequency of fast-food consumption as well as eating family meals in front of a television, and perceived stress.

**Conclusions:**

The present study found that poorer dietary choices, as well as other negative health-related impacts, occurred more often as the viewing time of a variety of different screen-based devices increased in a sample of US adults. Future research is needed to better understand what factors among different screen-based devices might affect health behaviors and in turn health-related outcomes. Research is also required to better understand how binge-watching behavior contributes impacts health-related behaviors and characteristics.

## Background

Television (TV) viewing represents one of the most common sedentary behaviors among the US population, with adults watching approximately 34 h per week [1]. Watching two or more hours of television per day has been associated with adverse health behaviors [2], numerous chronic diseases [3–5], cancer-related mortality and early mortality generally [3, 6]. Television viewing is also a significant predictor for diabetes diagnosis, abnormal glucose metabolism, hypertension, heart disease, high cholesterol, all-cause mortality, cardiovascular mortality, and cancer mortality [2]. Moreover, a graded association has been found between television consumption and health outcomes: adults watching television between 2.5 and 4 h per day were twice as likely to be overweight, and those watching television for more than 4 h per day were four times more likely to be overweight, compared to those watching less than 1 h per day [4].

In part, these outcomes might occur as a result of impacts of screen time on a variety of health-related behaviors, in particular dietary behaviors. Long hours of screen time have been associated with poor dietary patterns among adults, including higher consumption of sugar, especially from soft drinks; higher intake of foods with low nutritional quality like French fries, refined grain products, snacks and deserts; and lower intake of fiber, fish, vegetables, fruits, and whole grains [1–5]. Poor dietary choices may lead to overweight and obesity; for example, excess consumption of sugar-sweetened beverages has been found to be significantly associated with an approximate weight gain of 0.12–0.22 kg per year [6]. Conversely, healthier dietary patterns have been shown to be protective against a number of problematic conditions and diseases. Diets high in cereal fiber, for example, have been associated with a lower risk of developing type 2 diabetes independent of age, sex, and lifestyle factors [7], and a diet high in fruits and vegetables has been associated with a lower risk of cardiovascular disease and mortality [8, 9]. Hence, a dietary pattern marked by limited consumption of nutrient-dense foods may fail to confer optimal protection against chronic diseases, which could partially explain the association between prolonged television viewing and conditions such as type 2 diabetes and cardiovascular disease among adults [4, 10, 11].

Although associations between television time and health-related behaviors and outcomes is now well-established, the issue of screen time remains a complex and evolving problem. For example, even as Americans continue to consume multiple hours of TV per day, patterns of consumption are changing. Binge-watching, the continuous consumption of screen-based entertainment facilitated in part by media streaming services and television-connected devices, is growing as a phenomenon, potentially contributing to more evening-time screen use with the potential to crowd out other healthful behavioral goals and pursuits [12]. Simultaneously, the use of other sophisticated screen-based devices has grown considerably in recent years. In 2017, the use of ‘apps’ and internet services on a smartphone averaged more than 2 hours per day among American adults, contributing to a total average screen time use (television/television-connected devices/smartphone/tablet) of 8 hours daily for media consumption alone [13]. These changes could be important for dietary behaviors as well as other health-related behaviors and characteristics, including physical activity, sleep quality and duration, and stress [14].

Only recently has research begun to explore patterns of modern screen use across devices, times of day, and days of the week [15]. This approach to examining screen use represents an important next step in screen time research related to health. The combined used of multiple screens during a day can potentially impact health-related behavior in ways that are different than what has been seen in studies exploring TV use as the primary screen-based behavior. Further, it is not yet clear if and how the prolonged use of newer portable technologies, either alone or in combination, may be associated with poor dietary patterns that may further contribute to overweight, obesity, and other health-related outcomes. To the authors’ knowledge, no research exists exploring whether or how a variety of modern screen-based devices might be related to health behaviors differentially and collectively. The purpose of this study was to explore whether extended use of a variety of screen-based devices, in addition to television, was associated with dietary habits, physical activity, stress, sleep, and sleep quality among US adults.

## Methods

### Participants

A survey to assess screen time across multiple devices as well as dietary habits in a sample of US adults was administered using the Qualtrics platform and distributed via Amazon Mechanical Turk (MTurk). Data collected through MTurk, a crowdsourcing platform that allows researchers and companies to collect data from “virtual workers” in exchange for payment, has been shown to be valid and reliable in previous research [16].

Multiple strategies were followed to achieve high quality data (e.g. a “captcha” verification was included to exclude automated responses and spam, survey was only available to US MTurk workers, and responses were excluded if the worker failed the attention check in the middle of the survey, among others). The study was approved by the Institutional Review Board of Arizona State University.

Data collection was conducted during August 2019. Participants qualified for the study if they were: 1) adults 18 years of age and older, 2) English speakers, 3) current US residents, 4) owners of a television and at least one other device with a screen, and 5) noted that they watched television at least 2 h per day on most days. Eligible MTurk workers were asked to electronically sign an informed consent form and complete a survey through Qualtrics that asked about demographics, health characteristics, screen time habits, and usual eating practices. At the end of the survey, workers were provided with a completion code that allowed them to receive their payment through the MTurk website.

### Measures

#### Screen time

Screen time viewing time was assessed with an 18-item questionnaire that instructed respondents to estimate total time spent in hours and minutes using TV, TV-connected devices (e.g. devices that stream media content and video game consoles), laptops/computers, smartphones, and tablets. Respondents were asked “Thinking of an average weekday (from when you wake up until you go to sleep), how much time do you spend using each of the following types of screen as the primary activity?” For a more detailed description of this 18-item questionnaire please see Vizcaino et al. [15] The questionnaire has previously shown fair to excellent reliability, as assessed through intra-class correlation coefficients (ICCs = 0.50–0.90), and thus can be used to appropriately classify individuals into different screen time use categories (e.g. low vs. high) [15]. In addition, we explored the new phenomenon of binge-watching [16–18] by including the question “How often do you watch multiple episodes of a television program through digital streaming in a single sitting?” Participants were provided with five options ranging from 0 = never to 4 = daily.

#### Dietary habits

Dietary patterns were assessed with the Starting The Conversation (STC) tool, which is an eight-item food frequency instrument for use in primary care and health promotion [19]. The response items are organized into three different categories – most healthful dietary practices (0 score), less healthful dietary practices (1 score), and least healthful dietary practices (2 score). Item scores are added to create a summary score with a range 0 to 16; higher scores represent the greatest room for improvement. The STC has previously shown adequate psychometric properties [19].

Family meal frequency was assessed with the questions 1) “During the past seven days, how many times did all, or most, of the people living in your home eat a meal together without any screens on?” and 2) “During the past seven days, how many times did all, or most, of the people living in your home eat a meal together while watching television?” These were slightly modified from an original question used to examine the relationship between family dynamics, nutrition, and well-being [20]. Fast food intake was assessed with the question “In the past week, how often did you eat something from a fast food restaurant?” For all questions above participants answered in a Likert-type scale ranging from 0 = never to 7 = more than 7 times.

#### Other measures

Self-rated health was assessed with the question “How would you rate your health at the present time?” Participants answered in a Likert-type scale ranging from 1 = bad to 5 = excellent. Self-rated health has been found a strong and consistent independent predictor of mortality across a variety of populations in numerous studies [21]. Psychological stress was assessed with the 10-item version of the Perceived Stress Scale (PSS) [22], which measures the degree a person considers his life stressful in a Likert-type scale from 0 = never to 4 = very often. Physical activity levels were estimated with the Stanford Leisure-Time Activity Categorical Item (L-Cat) [23], which provides six descriptive categories of physical activity ranging from inactive to very active. Hours of sleep at night and overall sleep quality were estimated using questions from the Pittsburgh Sleep Quality Index (PSQI) [24]; sleep quality is rated in a Likert-type scale from 0 = very good to 3 = very bad. Lastly, body mass index (BMI) was calculated from participants self-reported height and weight. None of the questionnaires used required any permissions or licensing.

### Statistical analyses

Data were analyzed using the Statistical Package for the Social Sciences (SPSS) version 25.0. Variables under investigation violated assumptions of normality even after transformation attempts; hence, non-parametric tests were conducted. Descriptive data are presented as medians and interquartile ranges for continuous variables, and as proportions for categorical variables.

Three different screen time categories were created for total screen time. Similar to other research on screen time use [11], individuals falling in the lowest tertile for reported screen viewing time were categorized as “light users,” those in the middle tertile were categorized as “moderate users,” and those in the highest tertile were categorized as “heavy users.” The same procedure was repeated for screen time by type of screen (e.g. television, smartphone), except for tablet where “light users” and “moderate users” were combined into the same category due to a very small sample size in the “moderate users” category.

Kruskal-Wallis tests were conducted to examine differences in dietary habits between screen time categories. Significant results were followed up with pairwise comparisons using the Dunn’s procedure with a Bonferroni correction for multiple comparisons. Differences in demographics and health characteristics between screen time categories were examined also with Kruskal-Wallis tests for continuous variables, whereas chi-square tests were used for categorical variables. Spearman correlation was used to explore for associations between binge watching and variables under investigation. Significance was set at alpha < 0.05.

## Results

Nine hundred and seventy-eight responses were accepted from MTurk. Fifty-two participants were excluded for reporting greater number of screen time hours possible in a 24-h period for any given screen-based device. Hence, a total of 926 responses were included in the final analyses. Kruskal-Wallis tests indicated that participants were successfully assigned to significantly different total screen time categories; *X*^*2*^ (2) = 822.68, *p* < 0.000. Total screen time for light, moderate, and heavy users had a median of 7, 11.25, and 17.5 h per day, respectively. The only significant demographic differences between total screen time categories were age and race/ethnicity; *X*^*2*^ (2) = 10.32, *p* = .006 and *X*^*2*^ (8) = 28.26, *p* = .000, respectively. Heavy users tended to be slightly younger and have a greater proportion of African-Americans and Asians/Pacific Islander. See Table 1. Participants were also successfully assigned to significantly different screen time categories by type of screen (e.g. television, smartphone); all *p* < 0.000 (Table 2).

**Table 1:** Demographic characteristics of study participants

	*All participants* *(N = 926)*	*Light users* *(n = 296)*	*Moderate users* *(n = 311)*	*Heavy users* *(n = 319)*
Total screen time (minutes per day)^*^	690 (480–916.25)	420 (328.50–480)	675 (615–750)	1050 (900–1290)
Age^*^	34 (28–45)	34 (28–45)	35.5 (28–49)	32 (27–41.25)
Sex				
Male	44.9	42	45.8	46.5
Female	55.1	58	54.2	53.5
Race/ethnicity^*^				
Non-Hispanic White	72.7	78.2	76.8	63.7
Hispanic	7.7	7.1	7.7	8.2
African-American	8.6	4.8	6.5	14.2
Asian or Pacific Islander	8.4	7.8	6.5	10.7
Native American	2.6	2.0	2.6	3.2
Employment				
Full-time (≥ 35 h/wk)	63.5	60.0	61.9	68.2
Part-time (< 35 h/wk)	17.0	16.6	18.7	15.7
Not currently employed/retired	19.5	23.4	19.4	16.0
Income				
Less than 30,000	21.8	17.6	20.6	26.7
30,000 – 59,999	42.1	45.8	41.6	39.3
More than 59,999	36.1	36.6	37.7	34.0
Education				
Less than high school	0.5	0.3	0.3	0.9
High school/ GED/some college	36.4	35.9	37.2	36.0
Associate’s degree	12.1	12.5	12.3	11.4
Bachelor’s degree	37.4	34.2	38.2	39.4
Graduate or professional degree	13.7	16.9	12.0	12.3
Marital status				
Single (never married)	31.3	27.8	31.1	34.7
Married or in a committed relationship	60.5	66.1	60.2	55.5
Separated or divorced	6.4	4.4	6.8	7.9
Widowed	1.8	1.7	1.9	1.9

*Note.* Descriptive statistics are presented as medians and interquartile ranges for continuous variables and as proportions for categorical variables. Categories shown were created based on total screen time; light users ≤33.33th percentile, moderate users > 33.33th, 66.66th < percentiles, and heavy users ≥66.66th percentile. *Groups significantly different at alpha < 0.05

**Table 2:** Screen time by type of screen (minutes per day) reported by study participants

	*Light users*		*Moderate users*		*Heavy users*	
	Median (IQR)	N	Median (IQR)	N	Median (IQR)	N
TV^*^	60 (0–60)	222	120 (120–135)	372	240 (180–300)	327
TV-connected devices^*^	0 (0–0)	288	60 (60–60)	232	180 (120–240)	406
Laptops/computers^*^	60 (30–120)	292	240 (192.5–300)	274	480 (405–551.25)	360
Smartphone^*^	60 (22.50–60)	308	120 (120–120)	252	240 (180–360)	366
Tablet^*^	0 (0–15)	774	_	_	120.50 (120–240)	152

*Note.* Categories shown were created based on reported screen time viewing by device, except for tablet; light users ≤33.33th percentile, moderate users > 33.33th – 66.66th < percentiles, and heavy users ≥66.66th percentile. Tablet categories; light users ≤50th percentile and heavy users >50th percentile. *Categories significantly different from each other at alpha < 0.01

Descriptive data for dietary habits and health-related variables can be found in Table 3. Our results indicated that the dietary habits of heavy screen users significantly differed from the other two groups when analyzing by total screen time during the day. Dietary pattern scores were significantly higher in heavy users compared to moderate and light users indicating the least healthful dietary pattern; *X*^*2*^ (2) = 18.96, *p* = .000. Heavy users also reported the greatest number of days eating a meal together as a family while watching television (*X*^*2*^ (2) = 9.49, *p* = .009), and conversely, the least number of days eating a meal together as a family without any screens on (*X*^*2*^ (2) = 9.31, *p* = .009) as compared to moderate and light users. In addition, heavy users reported the highest frequency of fast food consumption compared to both moderate and light users; *X*^*2*^ (2) = 10.58, *p* = 005.

**Table 3:** Descriptive data for dietary habits and health-related variables between light-, moderate-, and heavy- users of screens by total screen time per day

	*Mean*	*SD*
Dietary habits variables		
Dietary patterns		
*Light*	5.45	2.26
*Moderate*	5.96	2.62
*Heavy*	6.33	2.58
Frequency of family meals without any screens on		
*Light*	2.80	1.52
*Moderate*	2.54	1.61
*Heavy*	2.51	1.52
Frequency of family meals while watching television		
*Light*	2.79	1.48
*Moderate*	2.85	1.61
*Heavy*	3.14	1.57
Frequency of fast food consumption		
*Light*	1.81	0.82
*Moderate*	1.86	0.80
*Heavy*	2.10	1.08
Health-related variables		
Body mass index (kg/m^2^)		
*Light*	25.44	6.23
*Moderate*	27.06	6.33
*Heavy*	27.00	7.95
Physical activity		
*Light*	2.21	1.41
*Moderate*	2.16	1.42
*Heavy*	1.85	1.38
Self-rated health		
*Light*	3.84	0.72
*Moderate*	3.79	0.66
*Heavy*	3.60	0.82
Hours of sleep		
*Light*	2.10	0.80
*Moderate*	2.05	0.84
*Heavy*	1.87	0.88
Sleep quality		
*Light*	2.86	0.70
*Moderate*	2.79	0.71
*Heavy*	2.70	0.81
Perceived stress		
*Light*	16.19	6.69
*Moderate*	16.11	7.08
*Heavy*	18.73	7.23

Interestingly, unique dietary habits emerged when examining dietary patterns by type of screen separately. For example, only heavy users of TV and smartphones reported the least healthful dietary patterns as compared to the other groups (*X*^*2*^ (2) = 13.02, *p* = .001 and *X*^*2*^ (2) = 20.23, *p* < .000, respectively); however, no significant differences in dietary patterns were observed between groups across TV-connected devices, laptop/computer, or tablet (*X*^*2*^ (2) = 2.63, *p* = .268; *X*^*2*^ (2) = .09, *p* = .954; *X*^*2*^ (2) = 1.66, *p* = .435). Consumption of fast food was consistently higher among heavy users of all screen devices (all *p* < .02), except for laptop/computer (*p* = .75). Please see Table 4.

**Table 4:** Mean ranks in dietary habits between light-, moderate-, and heavy- users of screens by total screen time per day and individual screen-based device

	*Total screen time per day*		*Television*		*Television-connected device*		*Laptop*		*Smartphone*		*Tablet*	
	Mean Rank	N	Mean Rank	N	Mean Rank	N	Mean Rank	N	Mean Rank	N	Mean Rank	N
Dietary patterns												
Light users	415.16	296	423.84	222	443.80	288	465.38	292	408.64	308	456.83	774
Moderate users	463.37	311	447.62	372	464.38	232	465.91	274	481.38^†^	252	–	–
Heavy users	508.48^*^	319	501.46ˆ	327	476.97	406	460.14	360	497.36ˆ	366	497.47	152
Frequency of family meals without any screens on												
Light users	501.56	296	472.22	222	472.30	288	478.75	292	484.98	308	456.99	774
Moderate users	445.46^†^	311	475.55	372	488.11	232	474.92	274	453.85	252	–	–
Heavy users	445.77^*^	319	436.83	327	443.19	406	442.44	360	452.07	366	496.66	152
Frequency of family meals while watching television												
Light users	442.04	296	410.49	222	466.03	288	461.31	292	447.27	308	463.87	774
Moderate users	446.49	311	420.70	372	426.17	232	468.52	274	454.10	252	–	–
Heavy users	500.00ˆ	319	541.14ˆ	327	483.04^**^	406	461.45	360	483.63	366	461.60	152
Frequency of fast food consumption												
Light users	435.51	296	430.11	222	404.77	288	472.35	292	420.16	308	453.65	774
Moderate users	454.33	311	455.55	372	496.05^†^	232	457.30	274	479.52^†^	252	–	–
Heavy users	498.41^*^	319	488.17^*^	327	486.56^*^	406	461.05	360	488.94 ˆ	366	513.67^*^	152

*Note*. ^*^Heavy users statistically different than light users at α < 0.05; ^**^Heavy users statistically different than moderate users at α < 0.05; ˆHeavy users statistically different than moderate and light users at α < 0.05; ^†^Moderate users statistically different than light users at α < 0.05

Heavy screen users also reported the lowest self-rated health (*X*^*2*^ (2) = 17.67, *p* = .000), the highest perceived stress scores (*X*^*2*^ (2) = 25.63, *p* = .000), and the least healthful behavioral patterns including the lowest number of hours of sleep (*X*^*2*^ (2) = 12.21, *p* = .002), poorest sleep quality (*X*^*2*^ (2) = 6.82, *p* = .033) and lowest amount of physical activity (*X*^*2*^ (2) = 13.56, *p* = .001). In addition, both heavy screen users and moderate screen users reported a significantly higher BMI as compared to light users (*X*^*2*^ (2) = 14.96, *p* = .001). Similarly, unique patterns emerged when examining by type of screen separately. For instance, physical activity was significantly lower only for heavy users of TV (*X*^*2*^ (2) = 11.39, *p* = .003), whereas self-rated health was significantly lower for heavy users of TV, TV-connected devices, and smartphones (*X*^*2*^ (2) = 6.84, *p* = .033; *X*^*2*^ (2) = 7.54, *p* = .023; *X*^*2*^ (2) = 10.52, *p* = .005; respectively) (please see Table 5).

**Table 5:** Mean ranks in health variables between light-, moderate-, and heavy- users of screens by total screen time per day and individual screen-based device

	*Total screen time per day*		*Television*		*Television-connected device*		*Laptop*		*Smartphone*		*Tablet*	
	Mean Rank	N	Mean Rank	N	Mean Rank	N	Mean Rank	N	Mean Rank	N	Mean Rank	N
Body mass index (kg/m^2^)												
Light users	411.99	295	441.37	220	439.48	287	447.14	289	430.93	307	459.90	769
Moderate users	489.27^†^	310	453.48	372	452.94	231	444.63	273	489.23^†^	252	_	_
Heavy users	479.02^*^	316	475.89	324	480.95	403	484.61	359	466.85	362	466.58	152
Physical activity												
Light users	490.16	295	506.63	222	459.36	288	458.87	292	478.94	308	467.25	774
Moderate users	480.44	311	457.84	371	484.85	231	462.54	272	462.09	251	_	_
Heavy users	419.29ˆ	318	430.70^*^	326	451.99	405	465.41	360	448.91	365	438.01	150
Self-rated health												
Light users	495.95	296	475.05	222	487.69	288	471.16	292	498.91	308	464.68	774
Moderate users	478.67	311	477.04	372	475.61	232	484.83	274	454.59	252	_	_
Heavy users	418.60ˆ	319	433.21^**^	327	439.42^*^	406	441.81	360	439.83^*^	366	457.48	152
Hours of sleep												
Light users	486.60	295	471.67	222	491.22	288	470.21	292	486.33	308	471.85	774
Moderate users	479.86	311	475.60	371	463.80	231	465.47	272	465.43	251	_	_
Heavy users	423.17ˆ	318	434.30	326	441.34^*^	405	454.00	360	440.38	365	414.28^*^	150
Sleep quality												
Light users	487.59	295	451.80	222	474.48	288	463.27	292	497.47	308	463.06	774
Moderate users	464.60	311	473.43	371	485.91	231	477.34	272	458.18	251	_	_
Heavy users	437.17^*^	318	450.31	326	440.63	405	450.66	360	435.97^*^	365	459.59	150
Perceived stress												
Light users	434.01	296	458.40	222	420.70	288	467.40	292	411.18	308	464.14	774
Moderate users	428.74	311	436.89	372	443.80	232	436.72	274	436.56	252	_	_
Heavy users	524.75ˆ	319	490.19^**^	327	505.12ˆ	406	480.72	360	526.08ˆ	366	460.25	152

*Note*. ^*^Heavy users statistically different than light users at α < 0.05; ^**^Heavy users statistically different than moderate users at α < 0.05; ˆHeavy users statistically different than moderate and light users at α < 0.05; ^†^Moderate users statistically different than light users at α < 0.05

Lastly, binge watching was significantly associated with least healthful dietary habits (*r =* .08, *p* = .02), frequency of fast food consumption (*r =* .13, *p* < .000), eating family meals in front of the television (*r =* .09, *p* = .008), and perceived stress (*r =* .18, *p* < .000). Please see Table 6.

**Table 6:** Correlation matrix of binge-watching and health-related variables

	Binge watching	Dietary patterns	Family meals without screens on	Family meals watching television	Frequency of fast food consumption	Body mass index	Physical activity	Self-rated health	Hours of sleep	Sleep quality	Perceived stress
Binge watching	1.00	0.08^*^	−0.05	0.09^**^	0.13^**^	0.02	−0.01	−0.04	−0.03	−0.04	0.18^**^
Dietary patterns		1.00	−0.03	0.09^**^	0.45^**^	0.12^**^	−0.26^**^	− 0.25^**^	− 0.13^**^	− 0.11^**^	0.20^**^
Family meals without screens on			1.00	−0.27^**^	0.08^*^	− 0.10^**^	0.14^**^	0.15^**^	0.09^**^	0.14^**^	−0.17^**^
Family meals watching television				1.00	0.05	0.09^**^	−0.04	−0.09^**^	−0.01	− 0.02	−0.13^**^
Frequency of fast food consumption					1.00	0.12^**^	−0.17^**^	−0.17^**^	− 0.06	−0.09^**^	0.21^**^
Body mass index						1.00	−0.18^**^	− 0.36^**^	−0.10^**^	− 0.13^**^	−0.10^**^
Physical activity							1.00	0.35^**^	0.08^*^	0.14^**^	−0.23^**^
Self-rated health								1.00	0.19^**^	0.29^**^	−0.32^**^
Hours of sleep									1.00	0.50^**^	−0.19^**^
Sleep quality										1.00	−0.36^**^
Perceived stress											1.00

## Discussion

The present study found poorer dietary habits among individuals spending a significant portion of their day using a variety of screen-based devices (i.e. total screen time). These “heavy users” reported the least healthy dietary patterns (e.g. they consumed few fruits/vegetables and regularly consumed sodas/sweet tea), the lowest frequency of meals shared with the family without screens on, and the highest frequency of fast food consumption. When analyzed separately by type of screen, only “heavy users” of television and smartphones showed statistically different scores in dietary patterns compared to the other groups. At the same time, both “heavy users” of TV and TV-connected devices reported a statistically higher frequency of family meals while watching TV, and “heavy users” of all devices except laptops showed a statistically higher frequency of fast food consumption. These results highlight the importance of separately exploring the impact of different screen-based devices on dietary habits instead of focusing only on an aggregate measure of total screen time.

Further, while frequency of fast food consumption, poor intake of fruits and vegetables, and excessive intake added sugars have all been connected to risk factors, and incidence of, chronic disease, the role of family meals is also important to health outcomes [6, 8, 9]. Research has identified frequency of family meals as a predictor of healthier dietary patterns and better weight management among children and adolescents [25]. Other work has shown that children who watched more television and also consumed fewer meals with the family were at greater risk of overweight [26]. Frequency of family meals may support improved family cohesion, problem-solving, and emotional coping as mediators of improved health outcomes [27], but it remains unclear if the potential of family meals to support more healthful outcomes for children and families could be disrupted by watching television or otherwise engaging in screen time during those meals. Given that heavy users of screens in our study reported the greatest number of days sharing a family meal while watching television as well as the highest intake of fast foods, more research is likely necessary to explore how simultaneous engagement in screen time and family meals might relate to the emotional and physical health of families.

More specifically, it may be possible that each screen-based device is associated with distinct factors that influence diet. For instance, TV viewing has been associated with poor dietary choices among children in part because of heavy advertising of candy, chips, sugary beverages, and fast foods [28, 29], and consequently advertisement may have a similar effect on food intake among adults watching TV [30]. Even so, while advertisements are embedded in social networks and free mobile apps, advertisement is less frequent in popular video-on-demand streaming services that can be accessed through an internet-enabled device, or TV-connected devices, as defined by this study.

Alternatively, associations found in this study between poor dietary habits and TV-connected devices may be related to the long hours of dedicated viewing that adults engage in through binge-watching, given that longer hours of TV-watching are associated with worse dietary patterns [1–5]. Such behavior might provide ample opportunity for the intake of multiple snacks of low nutritional value. Moreover, it has been suggested that the phenomenon of binge-watching differs from TV watching given the greater attentional focus required by complex narrative structures provided by video-on-demand shows [17], potentially interfering with conscious dietary choices. Our exploratory data supports this hypothesis; binge-watching was significantly associated with the least healthful dietary habits, frequency of fast food consumption, and eating family meals in front of the television.

The present study also found significant differences in other health variables among different screen time categories. Heavy users of all screens reported the lowest physical activity, self-rated health, hours of sleep, sleep quality, and highest perceived stress. Heavy and moderate users of all screens reported higher BMI scores compared to light users. Similar to dietary habits, unique patterns emerged depending on the type of screen analyzed; for example, “heavy users” of TV, TV-connected devices, and smartphones reported the poorest self-rated health compared to the other groups, whereas only “heavy users” of TV reported statistically lower physical activity compared to “intermediate” and “light users.”

Prior studies have found long hours of television viewing associated with premature mortality and cardiovascular disease (CVD) after adjusting for numerous covariates such as diet (e.g. energy intake, consumption of fruits/vegetables), physical activity, and sleep duration [3, 11]. It has been suggested that sedentary behavior may be primarily responsible for these adverse outcomes by decreasing the activity of lipoprotein lipase and subsequently affecting lipid and glucose metabolism [31, 32]. Our data indicate other potential explanatory factors that may mediate the relationship between extended television viewing – and other forms of screen time – and poor health. One such factor may be frequency of fast food consumption, which has been associated with poor diet quality [33, 34], and in turn has been associated with all-cause, CVD, and cancer mortality [35]. Other potential contributing factors include poor sleep quality, which has been shown to be related to disruptions in circadian rhythmicity [36, 37] and glucose metabolism [31]; and perceived stress, which has been shown to be significantly associated with adverse behaviors (e.g. smoking, drinking) related to CVD [38, 39]. In sum, our results indicate that prolonged screen time may be associated with a constellation of diverse factors that adversely impact health, perhaps differentially by type of screen.

While future research is needed to further characterize screen-time use across multiple screen-based devices, studies should also be conducted to better understand which factors across various devices might be associated with adverse health behaviors and in turn poor health-related outcomes either differentially or in combination. This line of inquiry is of priority especially for TV-connected devices given the increased popularity of video-on-demand services among Americans. A recent report indicated that 66% of the general population pays for a subscription of such services, and the percentage rises to 87% for households that consume more family-oriented movies and programming [40]. As the cultural phenomenon of binge-watching continues to gain momentum and acceptance, it is possible that increases in prolonged sedentary behavior coupled with poorer dietary choices could occur that could negatively impact overall health, especially among younger generations that are often more attracted to video-on-demand services [41]. On the other hand, an interesting new line of inquiry would be the potential benefits derived from moderate use of screens such as online learning and discovery, relaxation, and social connection [42]. One such study, for example, noted that media consumption could provide a boost to well-being, but only as long as other issues, such as a sense of guilt or the possible conflict between media consumption and pursuit of other goals, did not arise [43].

As with any study, important limitations existed in this work. The sample used to gather data for analyses was a convenience sample derived through MTurk; nonetheless, previous research has found that MTurk workers more closely resemble the US population compared to college samples and other internet samples [16]. Another important limitation is the nature of self-reporting, which may lead to underestimation of actual screen time use. Self-reporting has been characteristic of screen time cross-sectional research [44, 45], but with the advent of new technologies such as smartphone apps and screen time manager for TV, future studies would be able to use more objective assessments of screen time use. Further, as a cross-sectional study, it is not possible to verify directionality of the significant relations identified in this research. However, the unique findings of this work are strongly suggestive of the need for more studies that can further differentiate the ways health behaviors and health outcomes could vary by screen type and duration of screen use.

## Conclusion

Poorer dietary habits seem to be associated with extended use of screen-based devices, particularly television and smartphones. Physical activity, sleep and sleep quality, and stress might also be affected in different ways by different types of screen-based devices. Because the landscape of screen use in the US continues to evolve towards ever more complex configurations of usage and time commitments, future studies should continue to investigate how various screen-based devices might affect health behaviors and subsequently health-related outcomes in the long-term. Research may also incorporate considerations in relation to the impact of major life disruptions, such as the recent COVID-19 pandemic, which could greatly alter utilization of screens, potentially for better or worse. Further, it may be important to explore strategies for managing different types of screen use depending on the health-related characteristic or outcome of interest.

## Data Availability

The datasets used and/or analyzed during the current study are available from the corresponding author on reasonable request.
